# The Overexpression of Myelin and Lymphocyte Protein (MAL) Downregulates MUC1 and Enhances Cisplatin Sensitivity in Non-Small Cell Lung Cancer Cells

**DOI:** 10.7150/jca.129125

**Published:** 2026-03-17

**Authors:** Ana Karina Saldaña-Villa, Bismarck Vázquez-Almazán, Catalina Flores-Maldonado, Juan Carlos Vizuet-de-Rueda, Elisa Natalia Oropeza-Durán, José Bonilla-Delgado, Enoc Mariano Cortés-Malagón, Rubén Gerardo Contreras, Luis Manuel Teran, Blanca Ortiz-Quintero, Roberto Lara-Lemus

**Affiliations:** 1 Departamento de Biomedicina Molecular e Investigación Traslacional, Instituto Nacional de Enfermedades Respiratorias Ismael Cosío Villegas, Mexico City 14080, MEXICO.; 2 Posgrado en Ciencias Biológicas, Unidad de Posgrado, Edificio D, 1er Piso, Circuito de Posgrados, Ciudad Universitaria, Coyoacán 04510, Mexico City, MEXICO.; 3 Departamento de Fisiología, Biofísica y Neurociencias Centro de Investigación y de Estudios Avanzados del Instituto Politécnico Nacional (Cinvestav). Mexico City, MEXICO.; 4 Departmento de Inmunogénetica y Alergia, Instituto Nacional de Enfermedades Respiratorias Ismael Cosío Villegas Mexico City, 14080, MEXICO.; 5 Departamento de Biotecnología, Facultad de Ciencias de la Salud, Universidad Anáhuac México Campus Norte, Huixquilucan 52786, MEXICO.; 6 Laboratorio de la Unidad de Investigación en Oncología Molecular, Hospital de Alta Especialidad de Ixtapaluca, Servicios de Salud del Instituto Mexicano del Seguro Social para el Bienestar, Ixtapaluca 56530, MEXICO.; 7 División de Investigación, Hospital Juárez de México 07760, Mexico City, MEXICO.

**Keywords:** Myelin and lymphocyte protein, MUC1, lung cancer, cisplatin resistance

## Abstract

The epigenetic repression of myelin and lymphocyte protein gene (*MAL*) and over-expression of MUC1 protein are two well-documented hallmarks of various carcinomas including lung cancer. The purpose of this work was to investigate whether ectopic expression of MAL could modify the proliferative activity of MUC1 in human lung adenocarcinoma HCC827 cells. We generated stable HCC827 cell lines, expressing GFP- or myc-MAL constructs, then, followed the expression of MUC1 by RT-qPCR and Western blot, and tested proliferation and sensitivity of those cells to cisplatin. Our results showed that ectopic expression of MAL nearly eliminated cellular levels of MUC1-C. This effect is primarily due to down-regulation of *MUC1* expression, as confirmed by RT-qPCR. Additionally, lysosomal-associated degradation of MUC1 may also contribute, as observed when the cells were treated with ammonium chloride and chloroquine. The reduced quantity of MUC1-C negatively affected both cyclin D1 and c-Myc, protein levels, which are linked to cell-proliferative signals involving MUC1-C. Furthermore, expression of MAL decreased the viability of HCC827 cells, and increased their sensitivity to cisplatin. MAL and MUC1 showed an antagonistic relationship in cancer cells, and these findings provide new insights with respect to the regulation of MUC1 and a better understanding of MAL as a potential tumor suppressor.

## Introduction

Myelin and lymphocyte protein (MAL), also named T-lymphocyte maturation-associated protein, is a proteolipid constituent of cholesterol and glycosphingolipid-enriched micro domains (GEMs) that have been implicated in the apical transport of proteins in epithelial cells 1,2. MAL expression was initially demonstrated to occur during T-cell maturation 3 and was also reported in myelin-forming cells 4,5, as well as polarized epithelial cells 6-8. Several reports have indicated that silencing of MAL by hyper-methylation of its promoter 9 is found in different kinds of carcinomas, such esophageal 10-13, gastric 14, lung 15, colon 16, head and neck 17,18, cervical 19,20, breast 21,22, and bladder 23. Conversely, it has also been described that higher MAL expression levels are associated with the development of some types of lymphoma 24-27 and ovarian cancer 28-30. In this sense, tumor suppressor properties of MAL have been described by at least two well-documented reports; the first one, through the induction of apoptosis via the Fas signaling pathway, and the second, across the inhibition of cell migration and metastasis by blocking the signal transducer and activator of transcription 3 (STAT3) phosphorylation 10,31.

MUC1 (mucin 1), is a heterodimeric membrane-bound mucin, found in the apical region of epithelial cells. However, MUC1 is over-expressed and abnormally glycosylated in several types of adenocarcinomas 32-35. The oncogenic activity of MUC1 primarily depends on the MUC1-carboxy-terminal subunit (MUC1-C), which interacts with various mediators of cell proliferation signaling pathways 33. Overexpression of MUC1 and the loss of the plasma membrane (PM) polarity in transformed cells, enable interactions and phosphorylation of MUC1-C by receptor tyrosine kinases (RTKs), such as epidermal growth factor receptor (EGFR) 34,36. The binding of galectin-3 to the extracellular domains of EGFR and MUC1 stabilizes their interaction 36, and it has been reported that MUC1-C can prevent the lysosomal degradation of EGFR 37, thereby extending EGF´s proliferative activity.

MUC1-C activates various pro-survival pathways, such as Ras, PI3K/Akt, and Wnt 38-41. In this context, MUC1-C is a potent oncogene that exhibits several features that enhance tumor growth and metastasis. Recently, Rajabi et al. described a new pro-oncogenic activity of MUC1-C, involving the downregulation of tumor-suppressor genes 42. Therefore, we wondered whether a functional relationship exists between MAL and MUC1. In this sense, Fanayan et al. identified MAL2, another member of the MAL family, as a partner of MUC1 in breast carcinoma cells 43. They suggested that, in those cells, MAL2 could deliver MUC1 to the basolateral region, thereby promoting the phosphorylation of MUC1-C by EGFR and other tyrosine kinases, such as c-Src. Our laboratory is studying the role of MAL as a tumor suppressor in lung cancer. We hypothesize that MAL, a membrane raft scaffold protein, might interfere with the cellular trafficking of MUC1 and potentially alter its effects on cell proliferation. In this paper, we present evidence indicating that the expression of MAL in the NSCLC cell line HCC827 promotes the downregulation of the MUC1 gene and could facilitate the lysosomal degradation of MUC1-C. Both effects negatively impact the proliferation factors, cyclin D1, and c-Myc.

## Materials and Methods

### Cells, plasmids, and transfections

The lung adenocarcinoma HCC827 cells were provided by Dr. Jose Sullivan Lopez at the National Institute of Respiratory Diseases, Mexico. Dr. Lourdes Gutierrez supplied HEK293 cells at the National Institute of Public Health, Mexico. Both the HCC827 and HEK293 cells were cultured under standard conditions (5% CO2 and 95% air at 37 °C) in RPMI 1640 high-glucose medium, and DMEM high-glucose medium (Life Technologies), respectively. The media were supplemented with 10% FBS, 50 U/mL of penicillin, and 50 µg/mL of streptomycin (Life Technologies). The constructs pcDNA3.1-Hygromycin/GFP-MAL and pcDNA3.1-Hygromycin/myc-MAL were obtained from Dr. Peter Arvan´s laboratory (University of Michigan). Dr. Rebecca Hughey (University of Pittsburgh) kindly supplied the construct pcDNA3.1-Hygromycin/hMUC1-22r. Transfections were conducted using two µg/mL of plasmid pre-mixed with Lipofectamine 3000 reagent (Invitrogen) at a 2:1 ratio in reduced-serum medium. Six-well plates were seeded with 0.75 × 10^6 cells and transfected after 24 hours. Stable HCC827 and HEK293 cell lines expressing GFP-MAL, myc-MAL and hMUC1-22r were selected and maintained with 200 µg/mL of hygromycin B (Invitrogen). Several clones expressing GFP-MAL were isolated after fluorescence assisted cell sorting (Fig. S1A).

### Western blotting (WB)

Sub-confluent cell cultures were washed with ice-cold phosphate-buffered saline (PBS), and whole-cell lysates were prepared using RIPA buffer (Tris-HCl 25 mM pH 7.4, NaCl 150 mM, EDTA 1 mM, sodium deoxycholate 2.5 mM, NP40 1%, sodium dodecyl sulfate 0.1% and protease inhibitor cocktail, Complete C, from Roche). Protein concentration was measured using the bicinchoninic acid method (Thermo Scientific). Proteins were separated by SDS-PAGE in a Mini-PROTEAN Tetra cell electrophoresis chamber (Bio-Rad), according to Laemmli 44. The samples were transferred to nitrocellulose membranes using a semi-dry transfer system (Bio-Rad). Membranes were incubated with appropriate primary antibodies: anti-MUC1-C antibody, EPR 1023 (Abcam) 1:5000, anti-GFP (Bio-Legend) 1:5000, anti-cMyc (Immunology Consultants Laboratory) 1:5000, anti-cyclin D1 Invitrogen) 1/500, anti-EGFR A-10, and anti-MAL E1 (Santa Cruz Biotech) 1:1000 and 1:50 respectively. Secondary antibodies were from Jackson Immuno Research. Detection was conducted using chemiluminescence (Super Signal Kit, Thermo Scientific). Images were captured and analyzed using a ChemiDoc instrument (Bio-Rad).

### Ammonium chloride and chloroquine treatment

HCC827 wild-type (wt) cells, and HCC827-GFP-MAL clone (18P) were seeded at a density of 0.75 × 10^6 cells per well on 6-well plates. After 24 hours, the media were re-placed with fresh medium containing 0 or 5 mM ammonium chloride or 20 μM chloroquine. The cells were then incubated for an additional 24 hours. Whole-cell lysates obtained were immediately processed for immunoblotting.

### Immunofluorescence

HCC827-wt and HCC827-GFP-MAL cells were cultured on coverslips, washed twice with ice-cold PBS, and fixed with 4% formaldehyde, then permeabilized with 0.1% Triton X-100 and blocked with 2% ultra-pure BSA in PBS (blocking solution). The primary antibody anti-MUC1-C was diluted 1:100 in blocking solution. The secondary antibody was Alexa 555-tagged (Thermo Fisher). Confocal microscopy images were captured using an SP8 microscope, equipped with a Plan-NeoFluar 63x NA 1.4 objective (Leica Microsystems). We acquired confocal stacks of 40-60 images and created the maximum projection for each.

### Cell proliferation and cisplatin assays

HCC827-wt and 18P cells were seeded at a density of 2 × 10^4 cells per well in 96-well plates and incubated for 24 hours with various concentrations of cis diamine- dichloroplatinum (II) (cisplatin) (Sigma-Aldrich). A set of cells without any drug treatment was allowed to grow for 72 hours to assess differences in proliferation due to MAL expression. Cell proliferation was determined using the XTT reagent (Roche) according to the manufacturer´s instructions.

### cDNA synthesis and quantitative reverse transcription polymerase chain reaction RT-qPCR) analysis

The SV Total RNA Isolation System Kit (Promega, Cat. No. Z3100) was used to extract total RNA from all samples. For quantitative real-time PCR (RT-qPCR), RNA was treated with DNase I to remove genomic DNA. One microgram of total RNA was reverse transcribed in a 20 µL reaction mixture using SuperScript II-Reverse Transcriptase (Invitrogen) and an oligo(dT) primer. Real-time PCR quantification was performed with a StepOne Real-time PCR system (Applied Biosystems). RT-qPCR was conducted following the Maxima Probe qPCR Master Mix (2X)/ROX protocol (Thermo Scientific). A “no DNA” template control was included in each run. The data from the RT-qPCR experiments were analyzed using the relative quantification method, or 2-ΔΔCT method, where the ΔΔCT value = ((CT1 Target - CT1 Reference) - (CT0 Target - CT0 Reference)) 45. The mean CT values for both the target and internal reference genes were calculated, and the target gene fold change was normalized to HsGADPH. The relative expression of the target gene was then normalized to the control sample, whit the the control cells' expression set to 1 (i.e., 18P). The primers used included: HsGAPDH (AF261085.1) Forward 5´-GTCTCCTCTGACTTCAACAGCG-3´, Reverse 5´-ACCACCCTGTTGCTGTAGCCAA-3´. HsMAL (NM_002371.4) Forward 5´-GCTGGGTGATGTTCGTGTCTG-3´, Reverse 5´-TGAGGCGCTGAGGTAAAAGA-3´; and HsMUC1 (NM_002456.6) Forward 5´-TCGTAGCCCCTATGAGAAGG-3´, Reverse 5´-CCACTGCTGGGTTTGTGTAA-3´. All experiments were performed in triplicate (technical replicates) from three biological replicates.

### Statistical analysis

The statistical analysis used an independent-samples t-test to compare the means between two groups, and the normality of variances was verified using Shapiro-Wilk's test. The t-test was used to determine whether the observed differences were statistically significant (p < 0.05).

## Results

### The ectopic expression of MAL reduces the levels of MUC1

**Figure 1:**
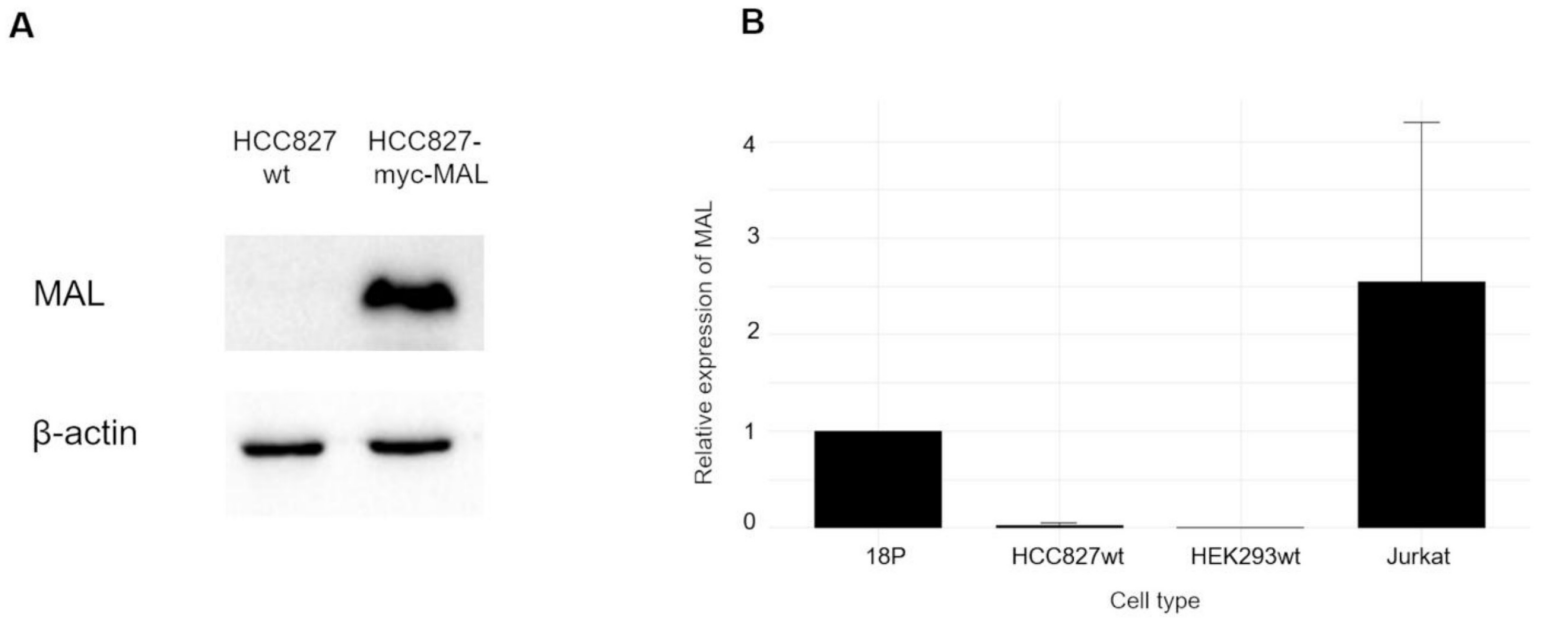
** Endogenous expression of MAL in HCC827-wt cells by WB and RT-qPCR. A.** The expression of MAL in cell lysates from cells HCC827-wt and HCC827 transiently transfected with the myc-MAL construct, were analyzed by WB. 20 μg of protein were loaded in each case. **B.** RT-qPCR analysis of HCC827-wt, HCC827-GFP-MAL-expressing (18P clone), HEK293 and Jurkat cells. Relative gene expression in control cells (18P) was defined as 1. HEK293 and Jurkat cells were included as negative and positive controls of the MAL expression respectively.

**Figure 2:**
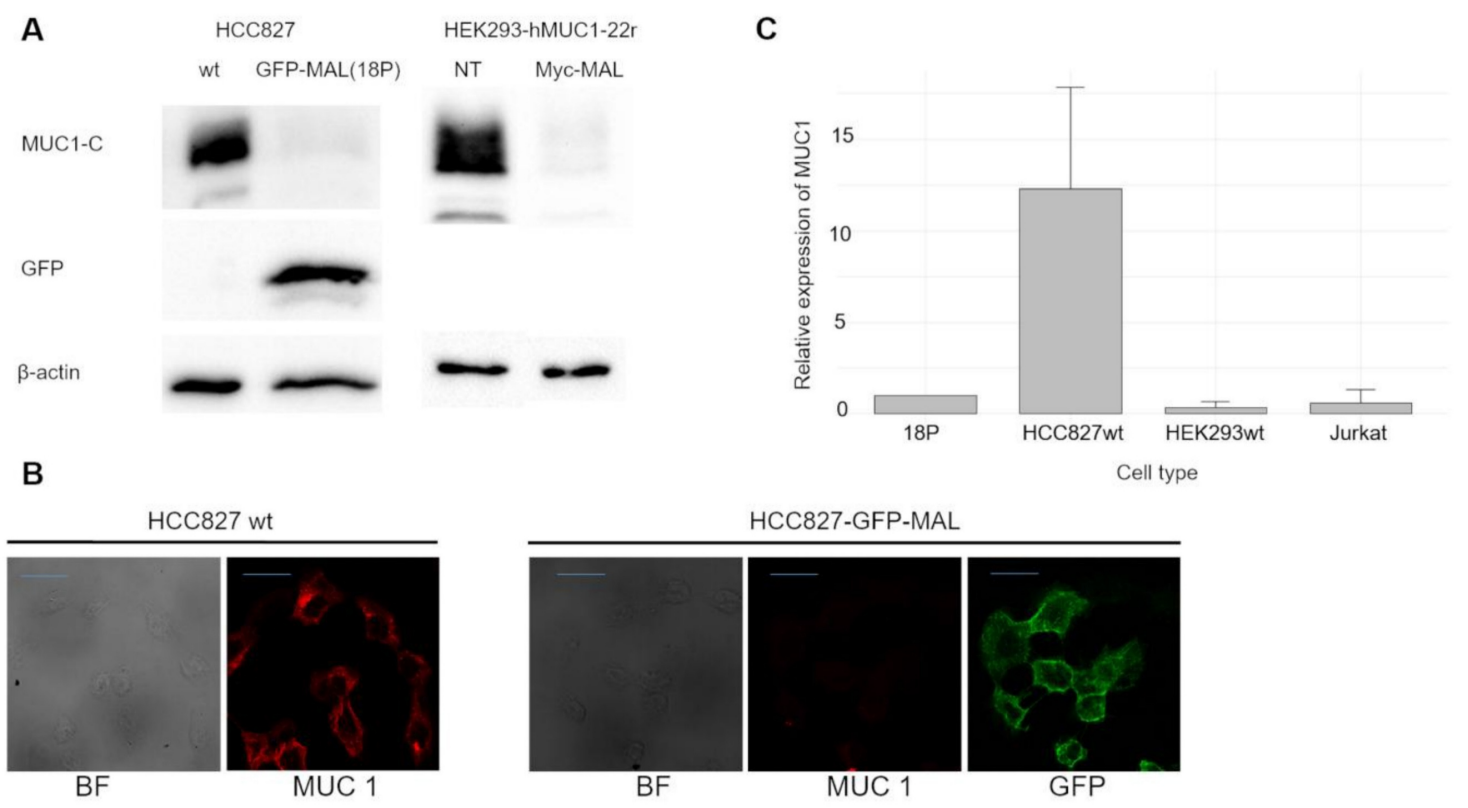
** The ectopic expression of MAL affect MUC1 expression. A.** WB of cell lysates from HCC827-wt,18P clone and HEK293-hMUC1-22r. This cell line was generated by the stable transfection of the human MUC1 cDNA, having 22 repeats of the variable number tandem repeat sequence (VNTR), described elsewhere 46, then, cells were transiently transfected with the myc-MAL cDNA. 20 μg of protein were loaded in each lane. **B.** Indirect immunofluorescence of HCC827-wt and HCC827 transiently transfected with GFP-MAL. MUC1, is in red. The decrease in the signal of MUC1-C agrees with the WB results. (bar = 20 μm). **C.** RT-qPCR analysis of *MUC1*-mRNA from 18P clone, HCC827-wt, HEK293 and Jurkat cells.

To our knowledge, no published data indicate that human lung carcinoma cells HCC827 express MAL. We began by evaluating endogenous MAL expression using WB and RT-qPCR. As shown in Fig. 1A, comparing the whole-cell lysate of HCC827-wt with that of cells transiently transfected with the myc-MAL construct, the non-transfected cells did not show any protein bands attributable to MAL. Furthermore, RT-qPCR analysis revealed only a negligible amount of MAL-mRNA in wt cells compared to the HCC827-GFP-MAL cells, (18P), as illustrated in Fig. 1B. This result aligns with published data for the other nine lung cancer cell lines 15. Next, as shown in Fig. 2A, WB analysis revealed that transfection with either GFP-MAL or myc-MAL significantly reduced MUC1-C levels in both HCC827-wt, and HEK293 cells stably transfected with human MUC1 (HEK293-MUC1h22r) 46,47. This cell line was transiently transfected with the myc-MAL construct, and WB was performed 48 hours after transfection. The result indicates that the phenotype is reproducible in HEK293-MUC1h22r, suggesting that the mechanisms induced by MAL expression are functional in cells that do not endogenously express MAL and MUC1. Fig. 2B displays the indirect immunofluorescence (IIF) results for HCC827 cells transiently transfected with the GFP-MAL construct. A significant reduction in the MUC1-C signal (red) can be observed in MAL-expressing cells, consistent with the WB. To confirm this, HCC827-GFP-MAL cells were sorted by flow cytometry, and several individual clones were isolated. WB analyses of whole-cell lysates revealed a significant decrease in MUC1-C levels in all MAL-expressing clones (see Fig. S1A).

### The expression of MAL reduces the amount of MUC1-RNAm and induces lysosomal degradation of the protein

To clarify why MUC1-C levels decrease in HCC827 cells upon MAL expression, we first examined whether MAL affects *MUC1* mRNA levels. Fig. 2C shows RT-qPCR results measuring *MUC1* mRNA in both HCC827-wt and 18P cells. In the presence of MAL, we observed a significant decrease in *MUC1* mRNA levels. While this could explain the substantial reduction in MUC1-C, we also investigated whether MAL is involved in degrading MUC1-C. It is known that MUC1 is primarily broken down in lysosomes, so we treated HCC827-wt and 18P cells with two lysosomal protease inhibitors. As shown in Fig. S1B, lysosomal-associated protein degradation with ammonium chloride or chloroquine partially restored MUC1-C levels in 18P cell lysates, indicating that MAL expression also promoted some degree of lysosomal degradation of MUC1-C.

### MAL expression affects the levels of cyclin D1 and cMyc

**Figure 3:**
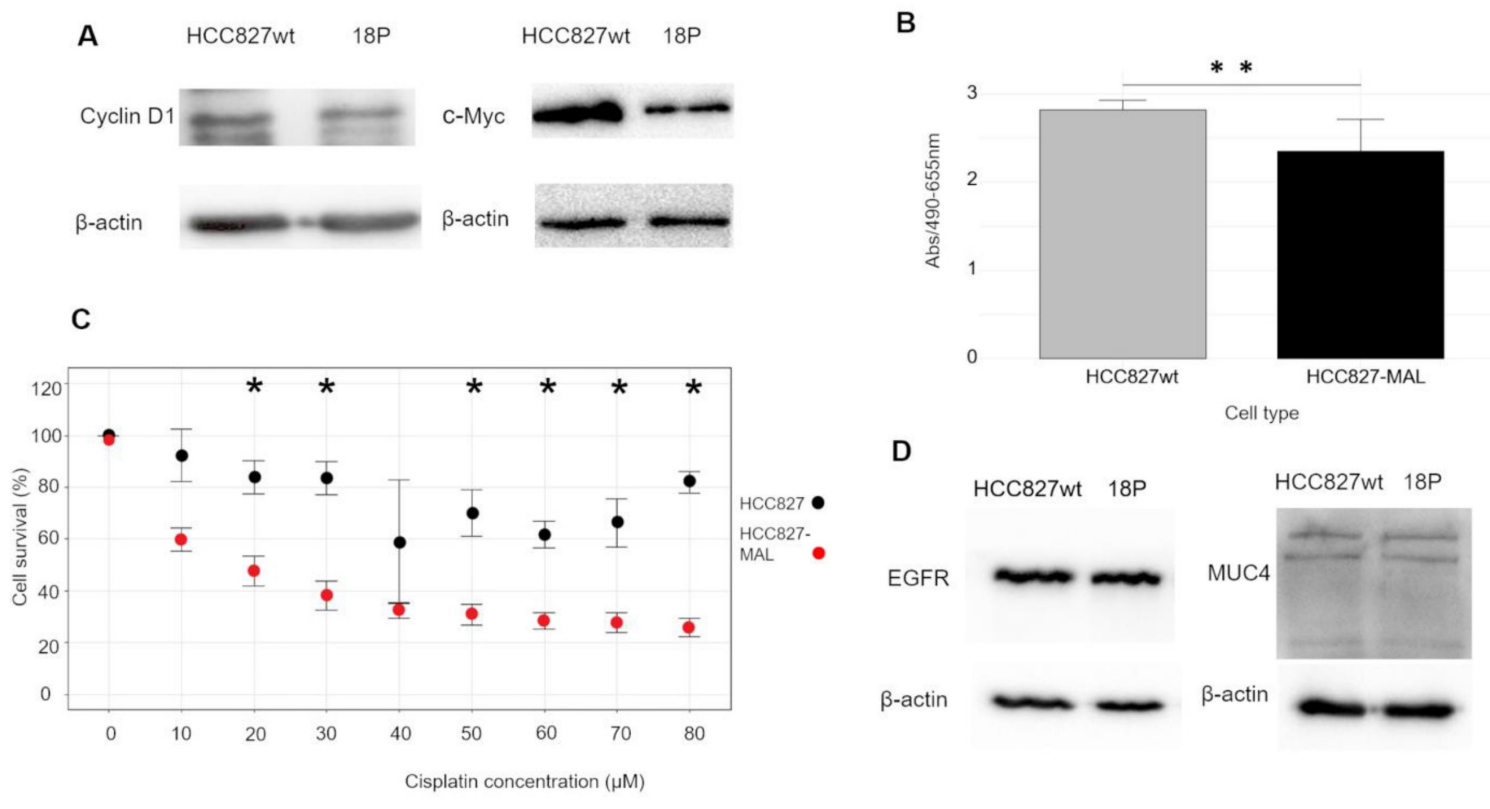
** Effects of MAL upon the expression of different proteins, proliferation and cisplatin resistance. A.** WB analysis of HCC827wt and 18P clone cell lysates for cyclin D1, and c-Myc; lower expression is observed in both cases when MAL is expressed. **B.** Proliferation of HCC827wt and 18P cells after 72 hours of culture. **C.** HCC827-wt (black dots) and 18P (red dots) were treated after 24 hours with different concentrations of cisplatin. Data represent mean ± SD from three independent experiments (n = 3). Statistical significance was determined with an unpaired two-tailed Student's t-test (*p < 0.05). **D.** EGFR and MUC4 WB of HCC827-wt and 18P cell lysates, no differences in the expression of both proteins were observed when MAL is expressed.

We examined whether MAL expression could affect the Wnt-MUC1-C relationship. β-catenin plays essential roles in processes such as cell-cell adhesion and transcriptional regulation. It binds to the sequence SAGNGGSSLS in the cytoplasmic tail of MUC1-C 48. In the nucleus, the MUC1-C-β-catenin complex activates the transcription of Wnt target genes 49 including *CCND1* and *c-Myc* 50-52. Therefore, we hypothesized that the decrease in MUC1-C caused by MAL would lower the expression of cyclin D1 (CD1) and c-Myc. As shown in Fig. 3A, WB analysis revealed a significant decrease in c-Myc and a slight reduction in CD1 levels in 18P cells. These findings support a tumor-suppressor role for MAL by interfering with the pro-oncogenic functions of MUC1-C.

### The ectopic MAL expression decreases the viability of HCC827 cells and increases their sensitivity to cisplatin

We hypothesized that decreased levels of CD1 and c-Myc might impact the proliferative capacity of HCC827 cells. To investigate this, we first compared the viability of HCC827-wt and 18P cells. As shown in Fig. 3B, the viability of 18P cells after 72 hours was significantly lower than that of the wt cells. This indicates that ectopic expression of MAL negatively impacts the growth of lung cancer cells. Next, we evaluated the response of HCC827-wt and 18P cells to the genotoxic agent cisplatin. Cells were exposed to increasing concentrations of cisplatin, and as shown in Fig. 3C, MAL-expressing cells exhibited lower survival and statistically significant differences at almost all cisplatin concentrations. The profile displayed by HCC827-wt cells showed a degree of cisplatin resistance, but MAL expression rendered the cells sensitive to the drug. Therefore, MAL expression hinders the proliferation of lung cancer cells.

### The effect of MAL seems to be specific to MUC1

To determine whether the MAL-induced phenotype was specific to MUC1, we examined its effects on other proteins. Like MUC1, EGFR is endocytosed through a mechanism involving clathrin-coated vesicles, recycled, and ultimately degraded in lysosomes 53-55. We investigated whether MAL expression could alter EGFR levels in 18P cells. As shown in Fig. 3D, the levels of EGFR were not affected by the ectopic expression of MAL. Additionally, we tested another membrane-bound mucin, MUC4, and again, no significant change in its cellular levels was observed in response to MAL (Fig. 3D). Therefore, the mechanism by which MAL reduces MUC1-C appears to be specific.

## Discussion

**Figure 4:**
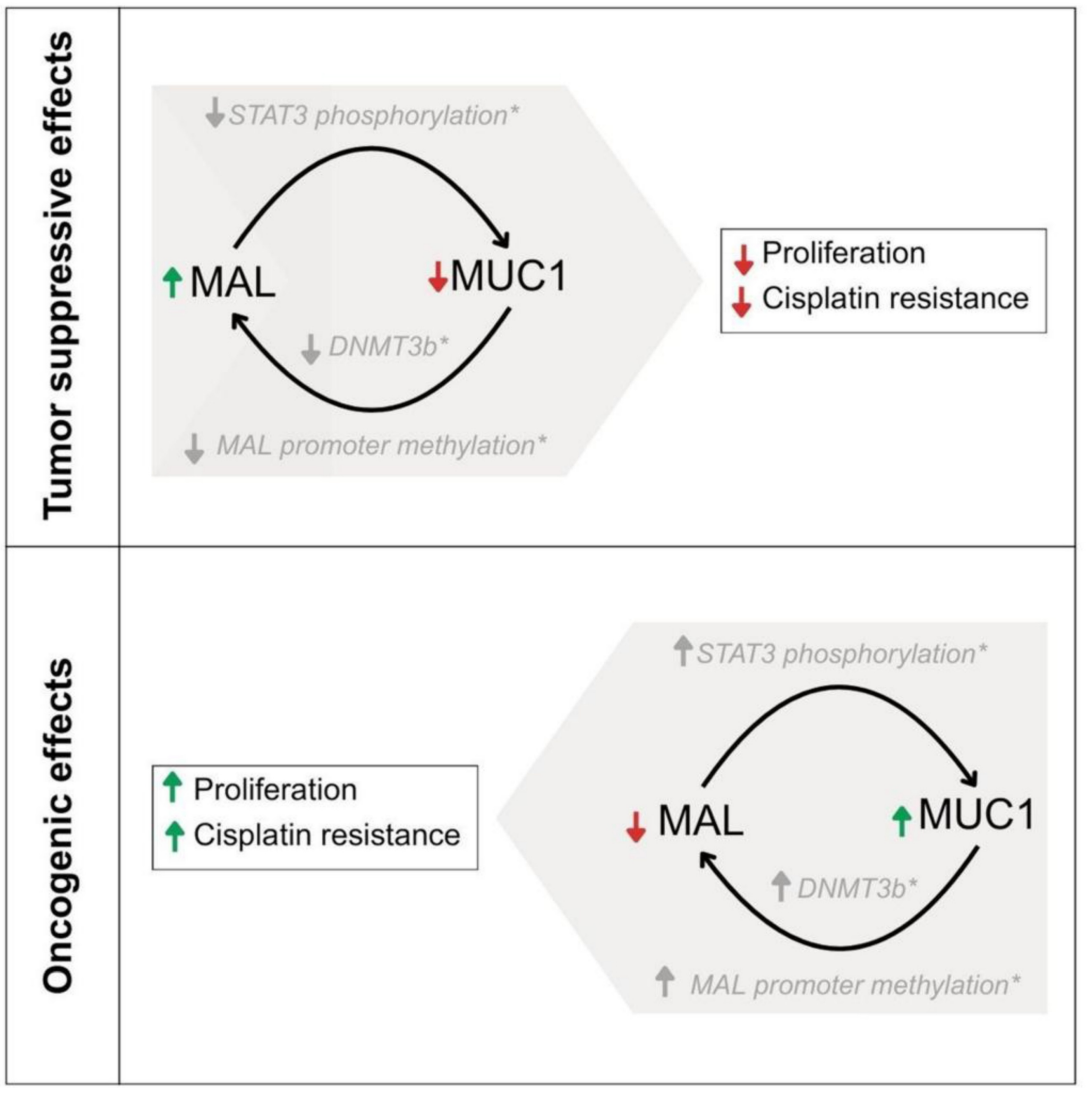
** A proposed model suggesting the possible explanation of the antagonist functions of MAL and MUC1-C.** The roles of STAT3 and DNMT3b are indicated in grey color as they were published previously by other researchers.

The down-regulation of MAL and over-expression of MUC1 have been observed in similar types of carcinomas 10-23, 32-35; however, no experimental evidence has previously demonstrated a direct effect of MAL, on the oncogenic activity of MUC1-C 56. In this study, MAL was undetectable in HCC827 cell-lysates by Western blot analysis (Fig. 1A). Indeed, MAL has been reported to be downregulated in nine other lung carcinoma cell lines through hypermethylation of its promoter, and its expression is restored after DNA demethylation with 5-Aza-2'-deoxycytidine 15. The results shown here reveal a strong correlation between ectopic *MAL*-cDNA expression and cellular levels of MUC1-C. Our data showed a significant decrease in MUC1-C levels in the HCC827-wt and HEK293-hMUC1-22r cell lines following transfection with either GFP- or Myc-MAL constructs. To explore the cause of this reduction, we examined both MUC1-C degradation and *MUC1*-mRNA levels. When lysosomal proteolytic activity was inhibited with ammonium chloride or chloroquine, we observed a slight recovery in cellular MUC1-C levels (Fig. S1B), suggesting that MAL may partially promote lysosomal degradation of MUC1-C. This finding is novel and the underlying mechanisms are unclear. In this regard, Razawi et al. 57 demonstrated that mutation of two specific tyrosine residues in the MUC1-C cytoplasmic tail, (Y20 and 60N) affects MUC1's clathrin-mediated endocytosis and causes the accumulation of MUC1 in endosomes. Our results suggest that MAL disrupts MUC1 recycling by directing it into the lysosomal compartment, even when signals for endocytosis and recycling are intact. Interestingly, it was reported that thyroid and breast cancer cells accumulate MUC1 in cytoplasmic vesicles, likely endosomes 58,59. From our immunofluorescence images (Fig. 2B), HCC827-wt cells also exhibit a cytoplasmic punctate pattern of MUC1-C, similar to that observed in breast and thyroid cancers. One possible explanation is that MAL may facilitate the transport of “stuck endosomes” loaded with MUC1 into lysosomes for degradation. The notable reduction in MUC1-C at the protein level cannot be explained solely by degradation. Therefore, we investigated *MUC1* gene expression. RT-qPCR results showed that MAL-expressing cells (18P) had lower *MUC1* mRNA levels (Fig. 2C). This is a significant and interesting finding. The* MAL* promoter is hypermethylated in several carcinomas 60, and it was demonstrated that MUC1-C activates the expression of DNA methyltransferases 1 and 3b (DNMT1 and 3b) 61,62. Indeed, the *MAL* gene is silenced by DNMT3b activity in NSCLC cell lines, including HCC827 63. On the other hand, previous reports have shown that ectopic expression of MAL reduces cell motility and tumorigenesis in esophageal carcinoma cells, likely by inducing apoptosis 10. More recently, Geng et al. reported that MAL interferes with the pro-oncogenic activity of STAT-3 in gastric cancer cells, by blocking its phosphorylation 31. Additionally, the *MUC1* promoter contains a phosphorylated STAT-3 (pSTAT-3) responsive element 64. Inhibition of STAT-3 expression decreases cell motility and *MUC1* expression in breast cancer cells 65. Although the mechanisms by which MAL interferes with *MUC1* expression remain unclear, the reported relationship between MAL and STAT-3 may help explain why ectopic MAL expression downregulates *MUC1* in HCC827 cells. Meanwhile, MUC4 is also upregulated by pSTAT-3 66,67, but we did not observe any decrease in MUC4 protein levels attributable to MAL expression (Fig. 3E). If MAL influences *MUC1* expression via pSTAT-3, this should be confirmed through future experiments. In cancer biology, our findings suggest that *MAL* plasmid expression counteracts the proliferative effects of MUC1-C and enhances cell sensitivity to cisplatin (Fig. 3C and D). Targeting MUC1-C with the cell-penetrating peptide GO-203, has been shown to inhibit cell proliferation, reverse cisplatin resistance, and reduce migration and invasion in esophageal carcinoma cells 68. In our study, we did not observe significant differences in invasion and cell migration between HCC827-wt and MAL-expressing cells (not shown). Lastly, we could not demonstrate a direct interaction between MAL and MUC1 via co-immunoprecipitation (not show), but Fanayan et al. using the yeast two-hybrid system, found that MUC1 is a partner for MAL-2, and that MAL can bind to MUC1 43. Therefore, we cannot dismiss the possibility of direct or indirect interactions between these proteins, which warrants future investigation. To conclude, this study provides the first experimental evidence that MAL reduces the MUC1-C levels in cancer cells, demonstrating an antagonistic relationship between MUC1 and MAL. This relationship results in a decreased proliferative capacity driven by MUC1-C and increased sensitivity to cisplatin. A proposed model explaining this antagonism is shown in Figure 4.

## Supplementary Material

Supplementary figure.

## References

[1] Millan J, Puertollano R, Fan L (1997). The MAL proteolipid is a component of the detergent-insoluble membrane subdomains of human T-lymphocytes. Biochem J.

[2] Martin-Belmonte F, Puertollano R, Millan J (2000). The MAL proteolipid is necessary for the overall apical delivery of membrane proteins in the polarized epithelial Madin-Darby canine kidney and Fischer rat thyroid cell lines. Mol Biol Cell.

[3] Alonso MA, Weissman SM (1987). cDNA cloning and sequence of MAL, a hydrophobic protein associated with human T-cell differentiation. Proc Natl Acad Sci USA.

[4] Kim T, Fiedler K, Madison DL (1995). Cloning and characterization of MVP17: a developmentally regulated myelin protein in oligodendrocytes. J Neurosci Res.

[5] Schaeren-Wiemers N, Valenzuela DM, Frank M (1995). Characterization of a rat gene, rMAL, encoding a protein with four hydrophobic domains in central and peripheral myelin. J Neurosci.

[6] Zacchetti D, Peranen J, Murata M (1995). VIP17/MAL, a proteolipid in apical transport vesicles. FEBS Lett.

[7] Millán J, Puertollano R, Fan L (1997). Caveolin and MAL, two protein components of internal detergent-insoluble membranes, are in distinct lipid microenvironments in MDCK cells. Biochem Biophys Res Commun.

[8] Martín-Belmonte F, Kremer L, Albar JP (1998). Expression of the MAL gene in the thyroid: the MAL proteolipid, a component of glycolipid-enriched membranes, is apically distributed in thyroid follicles. Endocrinology.

[9] Zorzan E, Elgendy R, Guerra G (2022). Hypermethylation-Mediated silencing of *CIDEA*, *MAL* and *PCDH17* tumour suppressor genes in canine DLBCL: from multi-omics analyses to mechanistic studies. Int.J. Mol. Sci.

[10] Mimori K, Shiraishi T, Mashino K (2003). MAL gene expression in esophageal cancer suppresses motility, invasion and tumorigenicity and enhances apoptosis through the Fas pathway. Oncogene.

[11] Wang Z, Wang M, Xu X (2000). Studies of MAL gene in human esophageal cancer by RNA *in situ* hybridization. Zhonghua Yixue Yichuanxue Zazhi.

[12] Kazemi-Noureini S, Colonna-Romano S, Ziaee AA (2004). Differential gene expression between squamous cell carcinoma of esophageus and its normal epithelium; altered pattern of mal, akr1c2, and rab11a expression. World J Gastroenterol.

[13] Jin Z, Wang L, Zhang Y (2013). MAL hypermethylation is a tissue-specific event that correlates with MAL mRNA expression in esophageal carcinoma. Sci Rep.

[14] Buffart TE, Overmeer RM, Steenbergen RDM (2008). MAL promoter hypermethylation as a novel prognostic marker in gastric cancer. Brit J Cancer.

[15] Suzuki M, Shiraishi K, Eguchi A (2013). Aberrant methylation of LINE-1, SLIT2, MAL and IGFBP7 in non-small cell lung cancer. Oncol Rep.

[16] Lind GE, Ahlquist T, Kolberg M (2008). Hypermethylated MAL gene a silent marker of early colon tumorigenesis. J Trans Med.

[17] Beder LB, Gunduz M, Hotomi M (2009). T-lymphocyte maturation-associated protein gene as a candidate metastasis suppressor for head and neck squamous cell carcinomas. Cancer Sci.

[18] Cao W, Zhang Z, Xu Q (2010). Epigenetic silencing of MAL, a putative tumor suppressor gene, can contribute to human epithelium cell carcinoma. Mol Cancer.

[19] Hatta M, Nagai H, Okino K (2004). Down-regulation of members of glycolipid-enriched membrane raft gene family, MAL and BENE, in cervical squamous cell cancers. J Obstet Gynaecol Res.

[20] Overmeer RM, Henken FE, Bierkens M (2009). Repression of MAL tumor suppressor activity by promoter methylation during cervical carcinogenesis. J Pathol.

[21] Guerrero-Preston R, Hadar T, Laskie-Ostrow K (2014). Differential promoter methylation of kinesin family member 1a in plasma is associated with breast cancer and DNA repair capacity. Oncol Rep.

[22] Horne HN, Lee PS, Murphy SK (2009). Inactivation of the MAL gene in breast cancer is a common event that predicts benefit from adjuvant chemotherapy. Mol Cancer Res.

[23] Blaveri E, Simko JP, Korkola JE (2005). Bladder cancer outcome and subtype classification by gene expression. Clin Cancer Res.

[24] Hsi ED, Sup SJ, Alemany C (2006). MAL is expressed in a subset of Hodgkin lymphoma and identifies a population of patients with poor prognosis. Am J Clin Pathol.

[25] Copie-Bergman C, Gaulard P.; Maouche-Chretien L (1999). The MAL gene is expressed in primary mediastinal large B-cell lymphoma. Blood.

[26] Copie-Bergman C.; Plonquet A, Alonso MA (2002). MAL expression in lymphoid cells: further evidence for MAL as a distinct molecular marker of primary mediastinal large B-cell lymphomas. Mod Pathol.

[27] Tracey L, Villuendas R, Ortiz P (2002). Identification of genes involved in resistance to interferon-alpha in cutaneous T-cell lymphoma. Am J Pathol.

[28] Schwartz DR, Kardia SLR, Shedden KA (2002). Gene expression in ovarian cancer reflects both morphology and biological behavior, distinguishing clear cell from other poor-prognosis ovarian carcinomas. Cancer Res.

[29] Berchuck A, Iversen ES, Lancaster JM (2005). Patterns of gene expression that characterize long-term survival in advanced stage serous ovarian cancers. Clin Cancer Res.

[30] Lee PS, Teaberry VS, Bland AE (2010). Elevated MAL expression is accompanied by promoter hypomethylation and platinum resistance in epithelial ovarian cancer. Int J Cancer.

[31] Geng Z, Li J, Li S (2022). MAL protein suppresses the metastasis and invasion of GC cells by interfering with the phosphorylation of STAT3. J Transl Med.

[32] Sousa AM, Grandgenett PM, David L (2016). Reflections on MUC1 glycoprotein: the hidden potential of isoforms in carcinogenesis. APMIS.

[33] Kufe DW, MUC1-C oncoprotein as a target in breast cancer (2013). activation of signaling pathways and therapeutic approaches. Oncogene.

[34] Bafna S, Kaur S, Batra SK, Membrane-bound mucins (2010). the mechanistic basis for alterations in the growth and survival of cancer cells. Oncogene.

[35] Irimura T, Denda K, Iida S (1999). Diverse glycosylation of MUC1 and MUC2: potential significance in tumor immunity. J Biochem.

[36] Kufe D (2009). Mucins in cancer: function, prognosis and therapy. Nat Rev Cancer.

[37] Pochampalli MR, el Bejjani RM, Schroeder JA (2007). MUC1 is a novel regulator of ErbB1 receptor trafficking. Oncogene.

[38] Li Y, Kuwahara H, Ren J (2001). The c-Src tyrosine kinase regulates signaling of the human DF3/MUC1 carcinoma-associated antigen with GSK3 beta and beta-catenin. J Biol Chem.

[39] Pandey P, Kharbanda S, Kufe D (1995). Association of the DF3/MUC1 breast cancer antigen with Grb2 and the Sos/Ras ex-change protein. Cancer Res.

[40] Raina D.; Kharbanda S (2004). and Kufe D, The MUC1 oncoprotein activates the anti-apoptotic phosphoinositide 3-kinase/Akt and Bcl-xL pathways in rat 3Y1 fibroblasts. J Biol Chem.

[41] Wen Y, Caffrey TC, Wheelock MJ (2003). Nuclear association of the cytoplasmic tail of MUC1 and beta-catenin. J Biol Chem.

[42] Rajabi H, Hiraki M, Kufe D (2018). MUC1-C activates polycomb repressive complexes and downregulates tumor suppressor genes in human cancer cells. Oncogene.

[43] Fanayan S, Shehata M, Agterof AP (2009). Mucin 1 (MUC1) is a novel partner for MAL2 in breast carcinoma cells. BMC Cell Biology.

[44] Laemmli UK (1970). Cleavage of structural proteins during the assembly of the head of bacteriophage T4. Nature.

[45] Livak KJ, Schmittgen TD (2001). Analysis of relative gene expression data using real-time quantitative PCR and the 2-ΔΔ*CT* method. Methods.

[46] Poland PA, Kinlough CL, Rokaw MD (1997). Differential glycosylation of MUC1 in tumors and transfected epithelial and lymphoblastoid cell lines. Glycoconjugate J.

[47] Lara-Lemus R, Saldaña-Villa AK, Vázquez-Almazán B (2018). Myelin and lymphocyte protein and mucin-1 in a transgenic expression model. Mens Bioquim.

[48] Huang L, Ren J, Chen D (2003). MUC1 cytoplasmic domain coactivates Wnt target gene transcription and confers transformation. Cancer Biol Ther.

[49] Li Y, Bharti A, Chen D (1998). Interaction of glycogen synthase kinase 3beta with the DF3/MUC1 carcinoma-associated antigen and beta-catenin. Mol Cell Biol.

[50] Rajabi H, Ahmad R, Jin C (2012). MUC1-C Oncoprotein Induces TCF7L2 transcription factor activation and promotes cyclin D1 expression in human breast cancer cells. J Biol Chem.

[51] Liu X, Caffrey TC, Steele MM (2014). MUC1 regulates cyclin D1 gene expression through p120 catenin and β-catenin. Oncogenesis.

[52] Bouillez A, Rajabi H, Pitroda S (2016). Inhibition of MUC1-C suppresses myc expression and attenuates malignant growth in KRAS mutant lung adenocarcinomas. Cancer Res.

[53] Tomas A, Futter CE, Eden ER, EGF receptor trafficking (2014). consequences for signaling and cancer. Trends Cell Biol.

[54] Madshus IH, Stang E (2009). Internalization and intracellular sorting of the EGF receptor: a model for understanding the mechanisms of receptor trafficking. J Cell Sci.

[55] Chi S, Cao H, Wang Y (2011). A novel form of the clathrin adaptor protein Eps15 mediates recycling of the epidermal growth factor receptor. J Biol Chem.

[56] Lara-Lemus R, On the role of myelin, lymphocyte protein (MAL) in cancer (2019). a puzzle with two faces. J Cancer.

[57] Razawi H, Kinlough CL, Staubach S (2013). Evidence for core 2 to core 1 O-glycan remodeling during the recycling of MUC1. Glycobiology.

[58] Ceriani RL, Chan CM, Baratta FS (1992). Levels of expression of breast epithelial mucin detected by monoclonal antibody BrE-3 in breast cancer prognosis. Int J Cancer.

[59] Bieche I, Ruffet E, Zweibaum A (1997). MUC1 mucin gene, transcripts, and protein in adenomas and papillary carcinomas of the thyroid. Thyroid.

[60] Rubio-Ramos A, Labat-de-Hoz L, Correas I (2021). The MAL protein, an integral component of specialized membranes, in normal cells and cancer. Cells.

[61] Rajabi H, Tagde A, Kufe D (2016). MUC1-C drives DNA methylation in cancer. Aging.

[62] Rajabi H, Tagde A, Alam M (2016). DNA methylation by DNMT1 and DNMT3b methyltransferases is driven by the MUC1-C oncoprotein in human carcinoma cells. Oncogene.

[63] Teneng I, Tellez CS, Picchi MA (2015). Global identification of genes targeted by DNMT3b for epigenetic silencing in lung cancer. Oncogene.

[64] Gaemers IC, Vos HL, Volders HH (2001). A stat-responsive element in the promoter of the episialin/MUC1 gene is in-volved in its overexpression in carcinoma cells. J Biol Chem.

[65] Yuan Z.L, Guan YJ, Wang L (2004). Central role of the threonine residue within the p+1 loop of receptor tyrosine kinase in STAT3 constitutive phosphorylation in metastatic cancer cells. Mol Cell Biol.

[66] Li G, Zhao L, Li W (2014). Feedback activation of STAT3 mediates trastuzumab resistance via upregulation of MUC1 and MUC4 expression. Oncotarget.

[67] Yang Y, Fang E, Luo J (2019). The antioxidant alphalipoic acid inhibits proliferation and invasion of human gastric cancer cells via suppression of STAT3-mediated MUC4 gene expression. Oxid Med Cell Longev.

[68] Zhao Y-Q, Wu T, Wang L-F (2021). Targeting MUC1-C reverses the cisplatin resistance of esophageal squamous cell carcinoma *in vitro* and *in vivo*. Transl Cancer Res.

