# Supplementary material for: The Overexpression of Myelin and Lymphocyte Protein (MAL) Downregulates MUC1 and Enhances Cisplatin Sensitivity in Non-Small Cell Lung Cancer Cells
Source: J Cancer. 2026 Mar 17;17(4):703–11. doi: 10.7150/jca.129125 (PMC13104723; doi:10.7150/jca.129125)
Supplement: Supplementary file 1 — Supplementary figure. [file jcav17p0703s1.pdf]

Fig. S1.

WB profiles of MUC1-C expression of different GFP-MAL expressing clones, and phenotype rescue by blocking lysosomal degradation of MUC1-C. A. Shows a typical WB of four different GFP-MAL expressing clones. Clones were isolated after fluorescence-assisted cell sorting (FACS). The drop in the MUC1-C amount is present in all clones. **B.** WB of whole-cell lysates of HCC8827-wt and the 18P clone, after 24 hours of incubation with ammonium chloride and chloroquine. A slight increase in the amount of MUC1-C was observed in 18P cells after the treatment.

Figure S1.

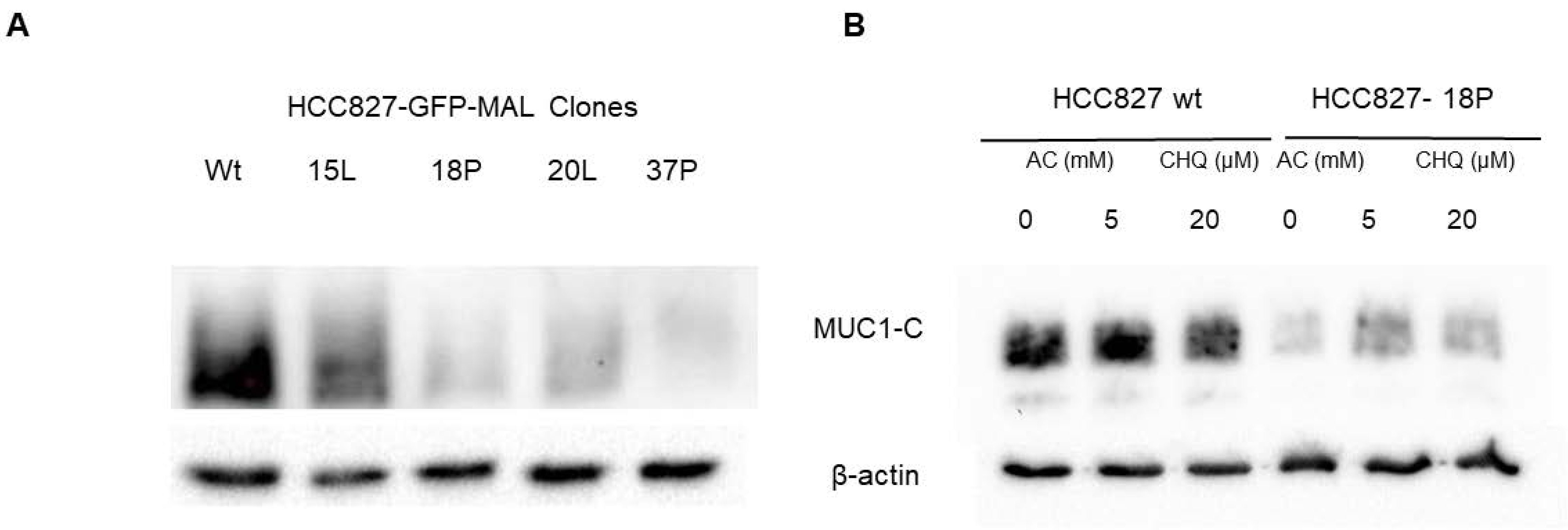
A
HCC827-GFP-MAL Clones
Wt
15L
18P
20L
37P
B
HCC827 wt
HCC827- 18P
AC (mM)
CHQ (μM)
AC (mM)
CHQ (μM)
0
5
20
0
5
20
MUC1-C
β-actin
